# Harnessing the power of social media in optimizing health outcomes

**DOI:** 10.11604/pamj.2014.18.290.4634

**Published:** 2014-08-14

**Authors:** Henry Nyongesa, Cecilia Munguti, Christopher Omondi, Winstar Mokua

**Affiliations:** 1 University of Nairobi, School of Medicine, Kenya

**Keywords:** Social media, health outcome, mobile phone

## Commentary

Since the advent of internet, there has been an explosion in the number of applications which have fundamentally revolutionized various sectors of life. The centerpiece of this communication advancement revolves around the position of social media [1–3]. Invariably, when social media is mentioned, many people associate it with Facebook, Twitter and You tube. However, social media comprises a myriad of online platforms ranging from micro blogs, blogs, social networking sites, wikis, video- and file- sharing, e-games, on line discussion forums, continuous professional education forums, research networking sites like research gate and information sharing sites like slide share, online training and seminars. The ease in accessibility and widespread use offers a cheap and easily available resource that can be harnessed in improving the health outcomes especially in developing countries. On the flipside, however, the variety of platforms elicits a variety of risks that negate these benefits. In medicine, particularly, the choice can be daunting since a wrong decision based on frivolous information may culminate in disastrous consequences. It is imperative for one to understand where most of their patients are deriving information to determine the reliability of the information.

### Benefits to patients and population

The effects of social media and internet generally are beyond imaginable proportions. In this generation, a great number of people go online to seek answers to general health questions. Patients’ quest for information concerning a specific disease, treatments, alternative treatment, medical insurance, healthcare providers and medical facilities and their capacity can be fulfilled by just a touch of a button[4–6]. In a USA survey by Mediabistro, more than 50% of respondents said that they made changes in management of prevention of diseases based on online readings [7]. Moreover, more than 40% of people said they were likely to change their decisions after seeking answers online. The greatest users of social media are young people, 90% of whom are likely to trust medical information shared through these networks. Online support group networks created purposely for patients suffering from particular conditions facilitate sharing of information. This concept of patient group networking provides social support in today's highly fragmented society. They also help patients understand their conditions and offer opportunities for them to better cope with their disease. Therefore, there is subsequent improvement in management of conditions, limited occurrence of complications, reduced rates of hospitalization, reduced cost of illness and reduced loss of income. Through this model, patients can be educated by a trusted healthcare forum about the signs, symptoms and conditions that warrant attention of a healthcare provider[8]. This model is remarkably revolutionizing patient management in most of Western countries. Additionally, social media can be a channel of marshaling the public to be aware about conditions which are either in epidemic or endemic proportion[9]. Government efforts in combating or mitigating a public health problem can be communicated via social media forums to the public. Besides the real time relay of information, the expenses involved are minimized. Additionally, the disease surveillance feedback programs can relay information via this media from the public to a central body. A faster response to the problem can then be initiated in case of a problem. People can also be informed about various government initiatives in improving their health welfare.

### Benefits to healthcare professionals

Though healthcare professionals are usually accused of their slow adoption of technology, the dynamics of patient care demand a paradigm shift from the traditional care model to the current model steered by social media[10]. The era of communicating disease information through booklets and pamphlets is almost being relegated to oblivion considering the ease, availability and minimal expenses involved when using social media forums. As a result of benefits reaped by being present on the platform, many health professionals are joining social media platforms [11]. Among the healthcare professionals, social media can promote the concept of crowd sourcing which though is controversial especially for highly sensitive areas like medicine, is the new fad in town[12]. Essentially, the concept revolves around harnessing the power of trusted healthcare workers on a given network to provide solutions to a given challenging clinical case the doctor is handling. While there is inherent fear of error in relying on some of the responses, the likelihood of zeroing in on appropriate management is increased. The issue of privacy may be a sensitive topic in the social media domain but in a survey by Mediabistro more than half of patients did not harbor any qualms against their care provider seeking answers of their medical condition through an online forum [7]. Interestingly, on some of the platforms, the practitioners post some interesting clinical vignettes from their practice for educative reasons[13]. This facilitates a wider body of knowledge on the disease. New products or drugs which have proven effective can also be explained appropriately to the HCPs through social platforms. Other online resources can be linked to such networks so that the members can acquire knowledge at their own time. Since there are a myriad of internet channels propagating various ways of managing conditions, healthcare professionals are obligated to generate educational content that will displace this misleading content[14, 15]. The techno savvy practitioners who run blogs can post comments that are beneficial not only among the patients but also towards fellow colleagues. Interesting articles that the author has come across can also be linked for other people to read. Microsoft's Dr. Bill Crounse proposition that blogging is the most effective way of promoting a health message adds impetus to creation of more blogs by professionals to fulfill this dictum. Google hangout platform provides a forum where the HCP can communicate or interact with his or her patients. This enables a better follow up on the patient conditions and institution of appropriate measures before complications set in.

### Benefits to health facilities

Increasingly, health institutions are claiming presence on the social media not only as a marketing platform but as well as providing information concerning available services. The forums can also be used to lodge any complaints concerning the quality of services [16–18]. The *Mayo clinic's* marketing strategy through the 3 social media sites is a success story that has been emulated across the US. Currently, there are more than 800 hospitals which have active presence on social media. Through their social media forum, patients get to download podcasts, videos and engage in chats in a chat room.

### Unresolved issues

Despite these immense benefits described, users and consumers of social media content should be wary of the inherent risks associated with unaccredited information. The web of information available through the outlets is so voluminous; it cannot lack obvious flaws which predispose users to bad and dangerous advice. In contention also is whether it is professional to use a forum that is laced with so much non medical activities for exchanging ideas among colleagues or with the patient. Additionally, there are various questions that emerge as regards patient's right to privacy and confidentiality when the case is shared among colleagues? Who vouches for the veracity of information relayed? Who will compensate the professional for engaging with the patient online? It is against such a background that institutions should come up with ways to compensate for this online consultation as well as limiting the risk of liability [19–21]. The greatest fear for any organization is controlling conversations on social media since some users may exploit such avenues to propagate negative publicity for the organization. It is therefore essential for health facilities to generate specific social media guidelines so that the staffs are on the same page and avoid the pitfalls of social media which can be used as channel for abusive content.

### Future of social media in the local healthcare setting

So what is the future of social media? We note that not many hospitals in the local health care setting are on the social media. This may stem from strict regulations on promotional and advertising on the medical profession[22]. Even the health care providers’ platform KMPDU is mostly used to disseminate political and litigations news rather than medical information. The patient community platforms are rudimentarily developed so that not many patients derive benefits from such sites. Most of content obtained is foreign based and may not fit in the local setting. On the etiquette front, not many institutions have put in place guidelines on usage of social media. For a robust social media presence, one has to be accessible and visible on the search. A clear description of the location, office hours, services and charges ought to be made to ease the consultation process. There is urgent need for patients to be informed of the existence of various media platforms that can be used to communicate with the providers, health facilities or obtain validated information on their conditions. A study conducted in US revealed that 86% of people aged 55-64 years don't use social media while a mere 24% don't in those aged 18-24. It is therefore imperative that the elderly Kenyan counterparts need to be encouraged to embrace the digital age trends as regards health service provision[23]. Health facilities and providers should also strive to engage their patients on social media forums so that the process becomes a two way. All these should be premised against a robust social media health care policy.

## Conclusion

In conclusion, social media not only accomplishes the mission of connecting doctors and their patients, but also enables patients to seek second opinion on decisions made. It also enhances social marketing of government and hospital services. While internet is no substitute for healthcare providers, it can enhance the depth of interaction between the clients and providers hence improving the health outcomes.

## References

[1] Abdul SS (2011). Facebook use leads to health-care reform in Taiwan. Lancet..

[2] Moorhead SA (2013). A new dimension of health care: systematic review of the uses, benefits, and limitations of social media for health communication. J Med Internet Res..

[3] Suby C (2013). Social media in health care: benefits, concerns, and guidelines for use. Creat Nurs..

[4] Househ M, Borycki E, Kushniruk A (2014). Empowering patients through social media: the benefits and challenges. Health Informatics J..

[5] Hopkinson NS (2014). Social media as a source of information for patients with chronic obstructive pulmonary disease. Chron Respir Dis..

[6] Timms C, Forton DM, Poullis A (2014). Social media use in patients with inflammatory bowel disease and chronic viral hepatitis. Clin Med..

[7] How healthcare pros are using social media. http://www.mediabistro.com/alltwitter/files/2012/08/social-healthcare.png.

[8] Queen D, Harding K (2014). Social media can revolutionise health care provider-patient relationship. Int Wound J..

[9] Mandeville KL (2014). Using Social Networking Sites for Communicable Disease Control: Innovative Contact Tracing or Breach of Confidentiality?. Public Health Ethics..

[10] (2012). Making “social” safer: are Facebook and other online networks becoming less hazardous for health professionals?. J Clin Ethics.

[11] Apostolakis I (2012). Use of social media by healthcare professionals in Greece: an exploratory study. Int J Electron Healthc..

[12] Adams SA (2011). Sourcing the crowd for health services improvement: The reflexive patient and “share-your-experience” websites. Soc Sci Med..

[13] Hamm MP (2013). Social media use by health care professionals and trainees: a scoping review. Acad Med..

[14] Knight E (2014). Physiotherapy 2.0: Leveraging Social Media to Engage Patients in Rehabilitation and Health Promotion. Phys Ther.

[15] Korda H, Itani Z (2013). Harnessing social media for health promotion and behavior change. Health Promot Pract..

[16] Greaves F (2014). Analysis of patients’ comments about hospitals in the english NHS via twitter, and comparison with patient surveys. BMJ Qual Saf..

[17] Greaves F (2013). Harnessing the cloud of patient experience: using social media to detect poor quality healthcare. BMJ Qual Saf..

[18] Thielst CB (2011). Social media: ubiquitous community and patient engagement. Front Health Serv Manage..

[19] Lachman VD (2013). Social media: managing the ethical issues. Medsurg Nurs..

[20] Social media (2014). What your hospital should know. Hosp Health Netw.

[21] Kind T (2014). Social media milestones: entrusting trainees to conduct themselves responsibly and professionally. J Grad Med Educ..

[22] Azizi T (2013). The issues surrounding social network sites and healthcare professionals. J Perioper Pract..

[23] Greave F (2012). Patients’ ratings of family physician practices on the internet: usage and associations with conventional measures of quality in the English National Health Service. J Med Internet Res..

